# A hybrid ResNet-ViT approach to bridge the global and local features for myocardial infarction detection

**DOI:** 10.1038/s41598-024-54846-8

**Published:** 2024-02-22

**Authors:** Junaid Abdul Wahid, Xu Mingliang, Muhammad Ayoub, Shabir Husssain, Lifeng Li, Lei Shi

**Affiliations:** 1 School of Computer and Artificial Intelligence, Zhengzhou University, Zhengzhou, 450001 Henan China; 2 School of Computer Science and Engineering, Central South University, Changsha, 410017 Hunan China; 3 School of Architecture, Harbin Institute of Technology, Shenzhen, 518055 Guangdong China; 4 Department of Radiology, The Affiliated Changsha Central Hospital, Hengyang Medical school, University of South China, Changsha, 410017 China; 5 School of Cyberspace and Security, Zhengzhou University, Zhengzhou, 450001 Henan China

**Keywords:** Biomedical engineering, Bioinformatics, Imaging, Biomedical engineering, Bioinformatics, Imaging

## Abstract

Myocardial infarction (MI) remains a significant contributor to global mortality and morbidity, necessitating accurate and timely diagnosis. Current diagnostic methods encounter challenges in capturing intricate patterns, urging the need for advanced automated approaches to enhance MI detection. In this study, we strive to advance MI detection by proposing a hybrid approach that combines the strengths of ResNet and Vision Transformer (ViT) models, leveraging global and local features for improved accuracy. We introduce a slim-model ViT design with multibranch networks and channel attention mechanisms to enhance patch embedding extraction, addressing ViT’s limitations. By training data through both ResNet and modified ViT models, we incorporate a dual-pathway feature extraction strategy. The fusion of global and local features addresses the challenge of robust feature vector creation. Our approach showcases enhanced learning capabilities through modified ViT architecture and ResNet architecture. The dual-pathway training enriches feature extraction, culminating in a comprehensive feature vector. Preliminary results demonstrate significant potential for accurate detection of MI. Our study introduces a hybrid ResNet-ViT model for advanced MI detection, highlighting the synergy between global and local feature extraction. This approach holds promise for elevating MI classification accuracy, with implications for improved patient care. Further validation and clinical applicability exploration are warranted.

## Introduction

Carotid artery plaque is a common manifestation of atherosclerosis, a chronic inflammatory disease characterized by the buildup of lipid deposits, inflammatory cells, and fibrous tissue in the walls of arteries^1,2^. This process can lead to the development of carotid stenosis, a condition characterized by the partial or complete blockage of blood flow through the carotid arteries and also cause for myocardial infarction (MI). Cardiovascular diseases, including MI, persist as a prominent cause of global morbidity and mortality. Myocardial infarction arises from blood flow obstruction within the heart muscle, resulting in irreversible tissue death^3^. Prompt and precise diagnosis is pivotal for effective MI management, as delayed treatment initiation can worsen heart muscle damage, elevating the risk of adverse outcomes, such as heart failure and mortality^4^. Despite advancements in medical technology and clinical practices, accurate myocardial infarction diagnosis remains a challenge. Misdiagnoses and identification inaccuracies concerning specific MI types can lead to inappropriate treatments, prolonging patient distress and increasing complications^5^. Traditional diagnostic methods, while partially effective, remain susceptible to subjectivity and discrepancies, especially in cases with atypical symptoms or patients with pre-existing cardiovascular conditions^6^.

Deep learning, a subset of artificial intelligence, is widely used in several other fields also, including image classification^7,8^, text analysis^9–11^, and situational awareness^12^. In addition, it is being implemented in agriculture to improve crop yield and management^13,14^. Apart from these, there are also various studies demonstrate the potential of deep learning-based approaches in improving the accuracy and efficiency of Cardiovascular diseases and ardiovascular image diagnosis^15,16^.

Several studies have embraced the capabilities of deep learning models to significantly enhance the detection of myocardial infarction through the analysis of electrocardiogram (ECG) signals. The study^17,18^ demonstrated the efficacy of convolutional neural networks (CNNs) in achieving cardiologist-level accuracy in arrhythmia classification, including myocardial infarction, underscoring the potential of deep learning in intricate cardiac diagnose. The ventured into predicting myocardial infarction presence using ECG data, achieving impressive accuracy in distinguishing between normal and abnormal ECGs, which opens doors for early detection of myocardial infarction^19^. Focusing on atrial fibrillation, the study^20^ harnessed deep neural networks to identify patients at risk, spotlighting the broader capability of deep learning models in detecting cardiac anomalies from ECG signals. In a similar vein, the development of a deep neural network capable of detecting and classifying arrhythmias, including myocardial infarction patterns, in ambulatory ECGs, presenting a powerful diagnostic tool^21^. Collectively, these studies exemplify how deep learning models, including CNNs and deep neural networks, can significantly elevate the accuracy of myocardial infarction detection, illuminating the transformative potential of AI-driven diagnostics in the realm of cardiac care.

However, despite the remarkable strides achieved by these studies, a significant challenge remains in the form of feature engineering. Crafting a robust feature vector that encapsulates the intricacies of myocardial infarction patterns is a complex endeavor^22^. While certain models, such as the Vision Transformer (ViT), have demonstrated the ability to capture global features and exhibit commendable performance, they come with the drawback of potentially overlooking crucial local features^23^. The ViT’s strength in comprehending the broader context might inadvertently lead to the underrepresentation of finer details that could be pivotal in accurate myocardial infarction classification. This conundrum is further accentuated by the existence of deep learning models that specialize in capturing local features while neglecting the global context. This dilemma underscores the intricacies in balancing the trade-offs between global and local feature extraction, hinting at the ongoing quest for an optimal fusion of these approaches to enhance the diagnostic accuracy and reliability of myocardial infarction detection. To tackle the robust feature vector challenge, we propose a fusion approach by combining ResNet and ViT feature vectors. During training, our method involves passing image data through both ResNet and ViT models. In ResNet, the last dense layer is removed to extract features from the last flatten layer (average pool). For ViT, we utilize the last hidden states from the last attention layer, flatten them, and employ an additional dense layer to align their dimensions with ResNet features. The merged feature sets are then processed through dense layers for final prediction. This novel strategy aims to synthesize global and local features for improved myocardial infarction classification accuracy. Our contributions in this study are elaborated below.

1. Overcoming limitations in the traditional ViT design by incorporating a slim model with a multibranch network and channel attention mechanism, enabling richer patch embedding extraction and improved learning capabilities.
2. Training image data through both ResNet and Modified ViT models, offering a dual pathway for feature extraction and encompassing a broader spectrum of features for classification.
3. By integrating global and local features, our approach addresses the challenge of robust feature vector creation, providing a more comprehensive representation of myocardial infarction patterns.

Through the integration of diverse features, our approach aims to achieve reliability in myocardial infarction detection, ultimately contributing to enhanced patient care and treatment decisions. The rest of the paper is organized as follows. The details of our approach are presented in “Section Materials and methods”. Section “Results and discussion” describes experimental evaluation results to validate the effectiveness of our approach. Finally, the conclusion and future work are drawn in “Section Conclusion”.

## Materials and methods

**Figure 1:**
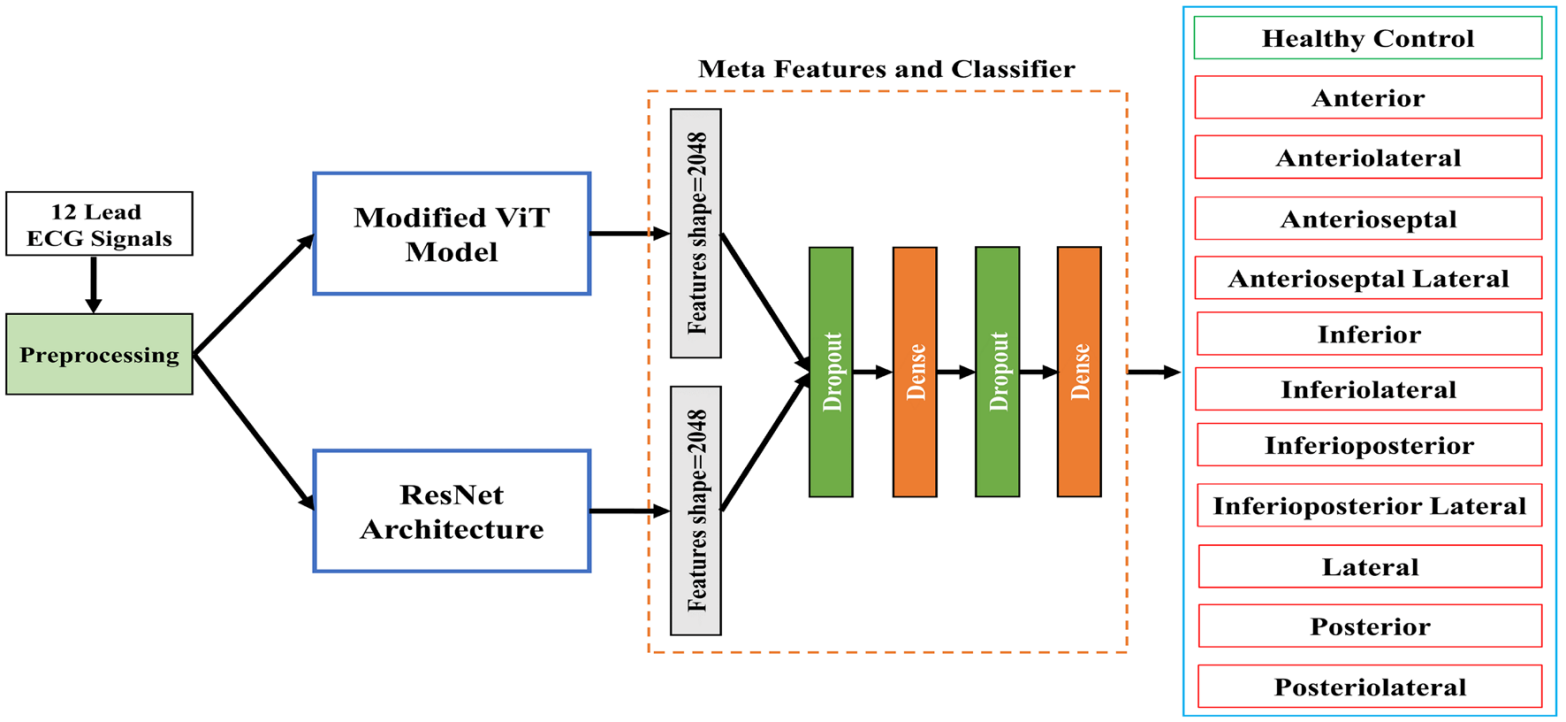
Proposed framework to classify 12 lead ECG for myocardial infarction detection.

In this section, we delineate our devised methodology tailored for myocardial infarction (MI) classification. We present a hybrid approach that combines a streamlined Vision Transformer (ViT) model, enriched with multibranch networks and channel attention mechanisms, and ResNet architecture to augment the model’s performance and elevate its accuracy in myocardial infarction detection. In our study, we utilized a pre-trained ResNet model with modifications for feature extraction. The ResNet model was optimized for the task by strategically removing the last dense layer. On the other hand, the hybrid Vision Transformer (HViT) slim model, introduced for feature extraction, was trained from scratch. Therefore, the ResNet model incorporated pre-trained weights up to the modified layer, while the HViT model was trained anew. This two-step approach allowed us to leverage the strengths of both architectures for a comprehensive representation of the input ECG image in the myocardial infarction dataset. The complete framework of our proposed study is shown in Fig. 1.

### Data collection

In this study the openly available dataset is used which is available on Mendeley repository (https://data.mendeley.com/datasets/gwbz3fsgp8/2). All methods were performed in accordance with the relevant guidelines and regulations. The ECG dataset used in our study comprises a substantial total of 1500 cases, intricately divided across different myocardial infarction (MI) types and a healthy control group. Among these, 120 cases belong to the “Healthy Control” group, providing a baseline for comparison. The remaining cases are meticulously distributed across various MI subtypes: “Anterior” (180 cases), “Anteriolateral” (145 cases), “Anterioseptal” (135 cases), “Anterioseptal Lateral” (125 cases), “Inferior” (170 cases), “Inferiolateral” (130 cases), “Inferioposterior” (155 cases), “Inferioposterior Lateral” (140 cases), “Lateral” (160 cases), “Posterior” (125 cases), and “Posteriolateral” (145 cases). The complete detailed statistics of the dataset is shown in Table 1.

**Table 1:** Statistics of the ECG dataset.

ECG type	Number of cases
Healthy Control	120
Anterior	180
Anteriolateral	145
Anterioseptal	135
Anterioseptal Lateral	125
Inferior	170
Inferiolateral	130
Inferioposterior	155
Inferioposterior Lateral	140
Lateral	160
Posterior	125
Posteriolateral	145

**Figure 2:**
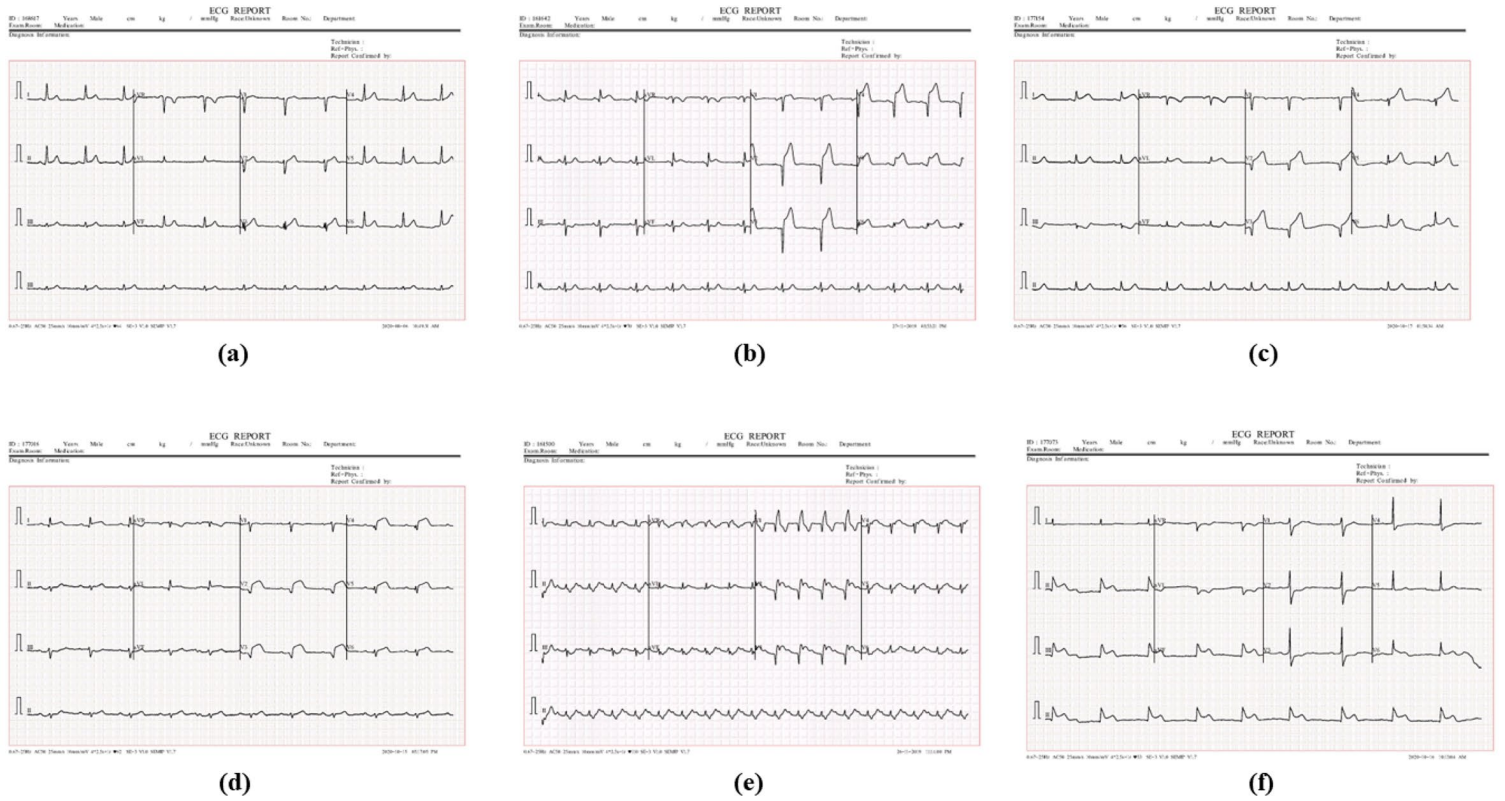
Sample images from ECG dataset.

The sample dataset is shown in Fig. 2. As we can see in Fig. 2a, the normal ECG with normal P waves, PR intervals, QRS complexes, ST segments and T waves, indicating no myocardial ischemia or injury. In Fig. 2b is anterior myocardial infarction, as the ST segment depression and pathological Q waves in leads V1–V6 indicating infarction of the anterior wall of the left ventricle. In Fig. 2c is the anterolateral myocardial infarction as the ST segment elevation in leads I and aVL with pathological Q waves indicating infarction of the anterolateral left ventricle. ST segment elevation and Q waves also present in leads V2–V5. The Fig. 2d is the anterior septal myocardial infarction. Pathological Q waves in leads V2–V3 indicating infarction of the anterior septum of the left ventricle. The Fig. 2e is the anterioseptal Lateral myocardial infarction. ST segment elevation in leads I and aVL with large pathological Q waves in leads V2–V3 indicating combined infarction of the anterior septum and anterolateral walls of the left ventricle. Similarly, Fig. 2f is the inferior myocardial infarction as ST segment elevation in the inferior leads II, III and aVF with reciprocal ST segment depression in leads I, aVL and V1–V3 indicating infarction of the inferior wall of the left ventricle.

The collected dataset verified and validated by two individual groups of domain experts who have ten years of experience in clinical ECG and cardiology and further their results also verified by one field expert to ensure the quality of collected dataset.

### Pre-processing of ECG

To address the preliminary segmentation challenges in ECG signals, we adopted an extensive pre-processing pipeline for enhanced clarity. The discrete wavelet transform (DWT) with the Daubechies 6 wavelet basis function^24^ was applied to effectively denoise the raw 12-lead ECG signals. The DWT is mathematically defined as in Eq. 1.

1 $$\begin{aligned} X(a, b) = \sum _{n} s(n) \cdot \psi _{a, b}(n) \end{aligned}$$

In Eq. 1$X(a, b)$ represents the wavelet coefficients, $s(n)$ is the original signal, $a$ and $b$ are the scale and translation parameters, respectively, and $\psi _{a, b}(n)$ is the wavelet function. This meticulous denoising step significantly reduced noise interference, enhancing the quality of the signals for subsequent analysis.

Following denoising, the Pan-Tompkins algorithm as shown in Algorithm 1 and 2^25^ was implemented for the precise detection of $R$ peaks, facilitating the accurate segmentation of heartbeats within the ECG signals. The automatic segmentation process resulted in individual heartbeats, each comprising a sample length of 651. Notably, each heartbeat was centered around the $R$ peaks, with 250 samples to the left and 400 samples to the right, ensuring comprehensive coverage of the cardiac cycle.

**Algorithm 1:**
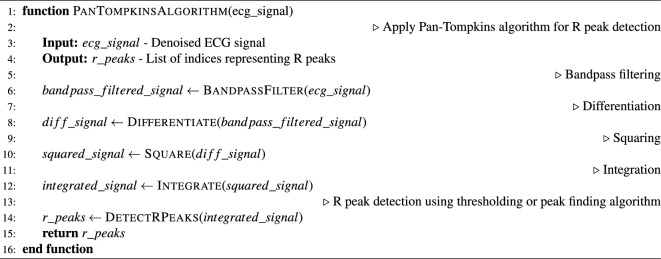
Pan-Tompkins Algorithm for R Peak Detection

**Algorithm 2:**
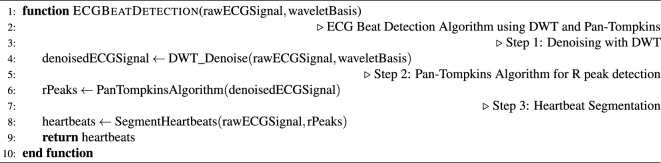
ECG Beat Detection Algorithm using DWT and Pan-Tompkins

In our proposed framework for heartbeat detection, the selection of the window size ($W$) is a critical parameter influencing the accuracy of QRS complex detection. We acknowledge the potential impact of T-wave variations and conducted a thorough analysis to mitigate their influence. The window size is chosen through the optimization process, aiming to capture the relevant features while minimizing the impact of T-wave variations. We introduce a cost function, $C(W)$, representing the trade-off between accurately detecting QRS complexes and reducing the interference from T-wave variations. The optimal window size is then determined by minimizing this cost function as shown in Eq. 2.

2 $$\begin{aligned} W_{\text {optimal}} = \arg \min _W C(W) \end{aligned}$$

Moreover, the relationship between the window size selection and the sampling frequency ($f_s$) of ECG records is crucial. A higher sampling frequency provides more detailed temporal information and allows for a finer granularity in the selection of the window size. We account for this relationship through the following Eq. 3.

3 $$\begin{aligned} W_{\text {optimal}} = k \times \frac{1}{f_s} \end{aligned}$$

In Eq. 3$k$ is a constant factor determined through empirical analysis. This mathematical representation elucidates the considerations made during the window size selection process and its explicit relationship with the sampling frequency, providing a formal framework for addressing potential challenges introduced by T-wave variations.

**Figure 3:**
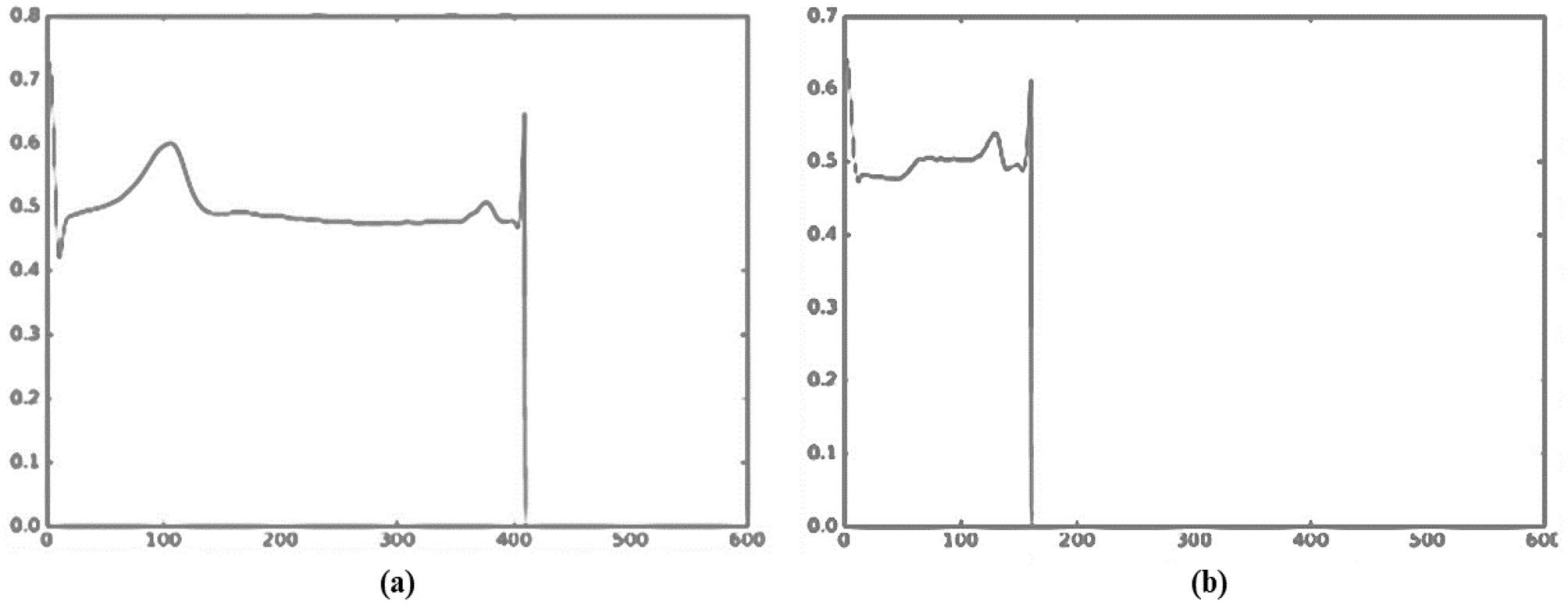
Sample ECG heartbeat after signal pre-processing and heartbeat segmentation. whereby (**a**) and (**b**) are the healthy and myocardial infarction respectively.

The sample image to see how the ECG processed and segmented can be seen in Fig. 3

Furthermore, for clarity and insight into the segmentation outcomes, Table 2 provides a detailed count of heartbeats for each class. This comprehensive approach to data pre-processing ensures robustness and accuracy in subsequent analyses.

**Table 2:** Number of heartbeats segmented for different classes.

Class	Number of heartbeats
HC	10,593
AMI	6470
ALMI	6691
ASMI	11,570
ASLMI	274
IMI	12,750
ILMI	8174
IPMI	49
IPLMI	2715
LMI	462
PMI	466
PLMI	787
Total	60,908

The combination of wavelet-based denoising and precise heartbeat segmentation ensures a robust foundation for subsequent analyses and contributes to the overall reliability of our study.

### ResNet-based feature extraction

In this section, we leverage the ResNet architecture for feature extraction. ResNet, short for Residual Network, is a deep convolutional neural network that has demonstrated remarkable effectiveness in various computer vision tasks, owing to its ability to mitigate the vanishing gradient problem and enable the training of very deep networks. Consider, *X* represent the input image, and $H_0$ be the input feature map obtained through the initial convolutional and pooling layers. The *i*-th residual block takes the input feature map $H_{i-1}$ and produces the output feature map $H_i$ as shown in Eq. 4.

4 $$\begin{aligned} H_i = H_{i-1} + F(H_{i-1}) \end{aligned}$$

In Eq. 4, *F* represents the residual function, which typically consists of multiple convolutional layers with batch normalization and activation functions. The ResNet architecture can be represented as shown in Eq. 5.

5 $$\begin{aligned} H_0 \xrightarrow {\text {Conv1}} H_1 \xrightarrow {\text {ResBlock1}} H_2 \xrightarrow {\text {ResBlock2}} \cdots \xrightarrow {\text {ResBlockN}} H_{N+1} \end{aligned}$$

In Eq. 5, $H_i$ denotes the feature map obtained after the *i*-th residual block, and *N* is the total number of residual blocks in the network.

To optimize the ResNet for our task, we strategically remove the last dense layer (fully connected layer) from the pre-trained ResNet architecture. This modification is motivated by the need to tailor the model for feature extraction, allowing us to harness features directly from the final flatten layer, which is typically an average pooling layer in ResNet. By doing so, we obtain a concise yet informative representation of the input data, crucial for preserving essential structural information within the complex ECG signals.

**Figure 4:**
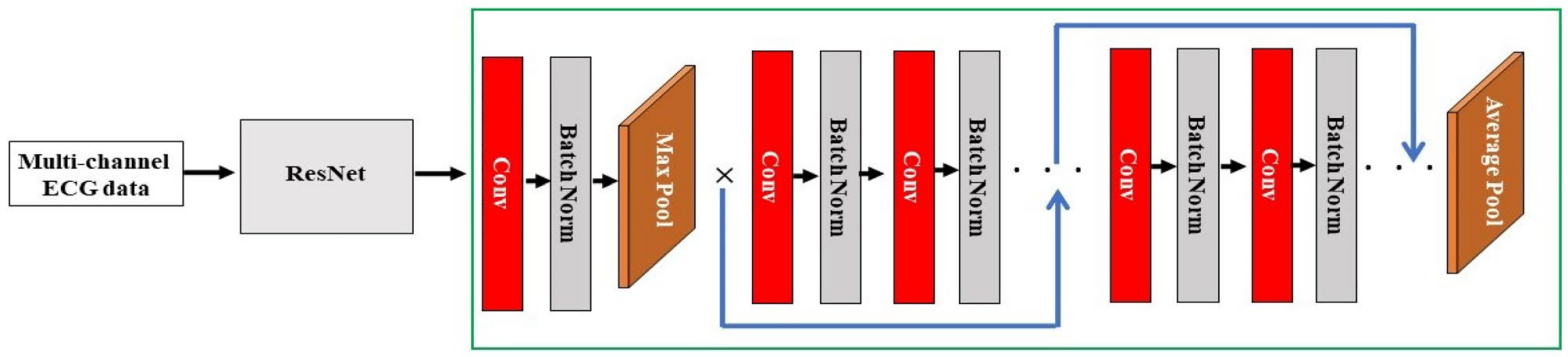
Complete architecture of ResNet for feature extraction.

The complete architecture of the ResNet utilized in our study is visually depicted in Fig. 4, providing an overview of its structure and connectivity. The model comprises initial convolutional layers, residual blocks, and concludes with the flatten layer. This architecture ensures that the ResNet operates as an effective feature extractor for subsequent classification task.

Additionally, Table 3 provides a detailed set of hyperparameters specific to our ResNet implementation. The hyperparameters include configurations for input layers, batch normalization, learning rate, convolutional layers, max pooling, initial filters, dropout rates, and weight decay. These parameters are carefully tuned to optimize the model’s performance for the classification of multi-channel ECG data.

**Table 3:** Complete hyper parameter details according to ResNet architecture.

Layer/param	Configuration/value
Input	Kernel: $3\times 3$, Stride: 1
BatchNorm	Momentum: 0.1, Epsilon:1e-5
Learning Rate	0.001
Conv	$3\times 3$, 1
Max Pooling	$3\times 3$, 2
Initial Filters	64
Dropout	0.2–0.5
Weight Decay	0.0001–0.001

The final feature vector obtained from ResNet is denoted as $F_{\text {ResNet}}$, and it serves as one of the inputs for subsequent processing steps. The combination of features from ResNet and ViT will be used for the final classification task, ensuring a comprehensive and robust representation of the input ECG image.

### Hybrid ViT for feature extraction

**Figure 5:**
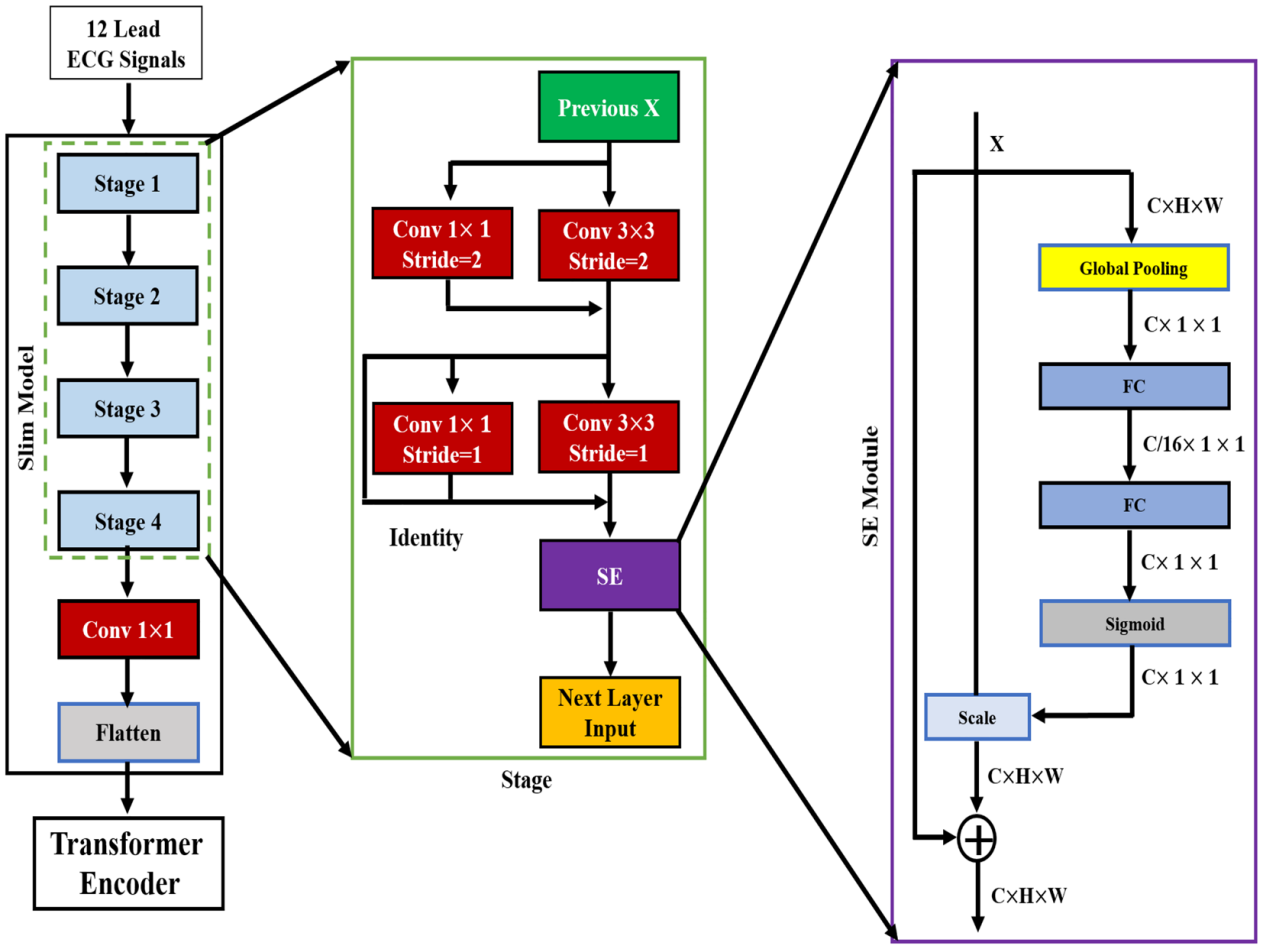
Proposed architecture by replacing the 16 × 16 convolution used in original ViT with a slimmer model.

**Figure 6:**
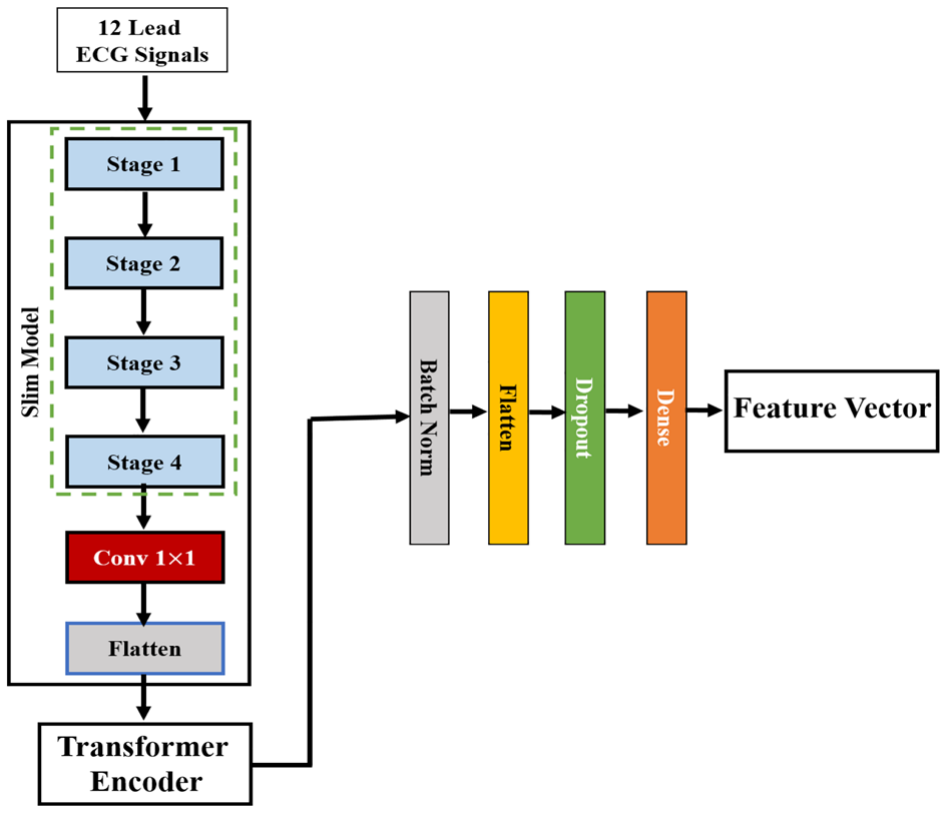
Overall HViT framework used in our study for feature extraction.

We introduce HViT as a modification to the original ViT model as shown in Figs. 5 and 6 where, we replace the 16 × 16 convolution used for image embedding extraction with a slimmer model. Specifically, we addressed the limitations of the traditional ViT model by incorporating a multibranch network and a channel attention mechanism, resulting in a slim model that enhances patch embedding extraction and enables the learning of richer information. This modified embedding is then passed to the subsequent transformer encoder. To maintain model efficiency, we reduce the number of transformer encoders by two, ensuring minimal changes to the overall model parameters and complexity.

As seen from the Fig. 5, the slim model is made up of a 1 × 1 convolutional layer and four stage modules. We utilize multiple small-scale convolutions instead of one large convolution in each step to improve the network’s diversity. The difference is that only simple 1 × 1 convolution and identity are used in the branching network, with the exception of the 3 × 3 convolution on the network backbone, which has the benefit of reducing network complexity and improving image feature reuse. We design the input and output of the slim model with reference to the embedding size extracted by the original ViT. Table 4 shows the architecture input and output specifications for the slim model. We are able to reduce the resolution of the feature map by half by setting the first branch network’s convolutional layer step to two.

**Table 4:** Input and output specifications for slim architecture.

Architecture	Input Size	Number of Channels
Stage 1	224	48
Stage 2	112	96
Stage 3	56	192
Stage 4	28	384
Conv 1 × 1	14	768

By utilizing four stages in HViT the output size can be reduced to 14 × 14, and the number of channels is increased to 768 through a 1 × 1 convolution. However, this approach may limit the model’s capacity to fully capture image-specific information, as ViT primarily aims to adapt the transformer architecture from NLP without extensive alterations. To address this problem, we used channel attention method, which assigns different weight coefficients to each channel, reinforcing essential features while suppressing non-important ones. In this article, a plug-and-play network module is employed for channel attention, as depicted in Fig. 5.

The module first transforms the input feature map into a vector using global average pooling, capturing the global distribution of channel responses. Two fully connected layers are then utilized to establish correlations between channels. The first layer reduces the feature dimension, while the second layer restores it to the original size. Finally, the Sigmoid function is applied to weight the previous features on a channel-by-channel basis, facilitating feature selection. In order to improve feature reuse within each stage and achieve better performance with limited network depth, we incorporated the identity concept within the SE model using the dense block idea. This allows for enhanced information flow across stages.

The entire operation of the stage is outlined in Eqs. 6 and 7.

6 $$\begin{aligned}{} & {} O(x)=F_{S=2}(x)+L_{S=2}(x) \end{aligned}$$

7 $$\begin{aligned}{} & {} H(x)=SE[F_{S=1}(O(x))+L_{S=1}(O(x))+O(x)] \end{aligned}$$

In Eqs. 6 and 7$F_{s=2}(\dot{)}$ indicates the 3 × 3 convolution of stride = 2, $L_{s=2}(\dot{)}$ means the 1 × 1 convolution operation of stride = 2, $F_{s=1}(\dot{)}$ denotes the 3 × 3 convolution of stride = 1, $L_{s=1}(\dot{)}$ denotes the 1 × 1 convolution operation of stride = 1, and SE[.] signifies the SE model operation. HViT has a more neural network compliant method for extracting Patch Embedding original ViT. On the one hand, the model improves the diversity of features contained in embedding using a multibranch structure. On the other hand, the channel attention mechanism is used to compensate for the disadvantage of not being able to obtain the importance of the image channel in ViT.

#### Feature engineering

In the Hybrid Vision Transformer (HViT) model, the last hidden states obtained from the last attention layer encompass information from all the image patches except the classification token; then we flatten them and use another dense to reduce the shape to make the output (features) have the same size as the feature extracted from ResNet. Ensuring parity in feature dimensions between the ResNet and Vision Transformer (ViT) architectures is imperative for coherent feature integration in subsequent model stages. The harmonization of feature dimensions is crucial to facilitate seamless merging, ensuring compatibility and alignment during the aggregation process. In cases where feature dimensions diverge, inconsistencies may emerge, leading to potential information loss, misalignment, or suboptimal model performance. The uniformity of feature dimensions, therefore, serves as a foundational principle for optimizing the compatibility and synergy of the extracted representations, contributing to an integrated ResNet and ViT model with enhanced predictive efficacy. The Fig. 6, represents the HViT which we used in our studay by addition of some flaten and dense layers to normalize the feature shape to same as ResNet.

The amalgamation of features from the ResNet and Vision Transformer (ViT) models involves a well-defined process to create a unified representation for subsequent processing. In the ResNet architecture, the last dense layer is removed from the pre-trained model, allowing for the extraction of features from the last flatten layer (average pool). Simultaneously, in the ViT model, the last hidden states are obtained, encompassing all patches from the last attention layer, excluding the classification token. These hidden states are then flattened, and another dense layer is employed to adjust the shape, ensuring the output features have the same dimensions as those extracted from ResNet.

The critical step in the amalgamation process is the concatenation of the features obtained from ResNet and ViT. This concatenation operation combines the distinctive characteristics captured by each model, creating a unified feature set that encapsulates the unique information learned by both architectures. The concatenated feature set is subsequently passed through a series of additional dense layers, enabling further refinement and integration of the complementary information derived from ResNet and ViT. This comprehensive operation enhances the synergy between the two models, culminating in a final prediction with improved predictive capabilities.

Mathematically, let’s denote the input image as $X$, and the tokenized image patches as $P_1, P_2, \ldots , P_N$, where $N$ is the total number of patches. Each patch is represented as a vector of features: $P_i = [p_{i1}, p_{i2}, \ldots , p_{id}]$, where $d$ is the dimension of the patch features.

The HViT model consists of a linear projection that maps the input patches $P_i$ to embeddings $E_i$: $E_i = W_e \cdot P_i + b_e$, where $W_e$ is the projection weight matrix, and $b_e$ is the bias term.

To obtain a single feature vector that captures the contextual information from all the patches, we flatten the hidden states and apply another dense layer: $F = W_f \cdot \text {Flatten}(H) + b_f$, where $\text {Flatten}(H)$ is the flattened hidden states matrix, $W_f$ is the reduction weight matrix, and $b_f$ is the reduction bias term. $F$ now represents the final feature vector, and its dimension is the same as the feature extracted from ResNet.

The amalgamation of features from ResNet and HViT involves concatenation and subsequent refinement through additional dense layers. This comprehensive operation enhances synergy between the two models, culminating in a final prediction with improved predictive capabilities. Mathematically, the HViT model’s operation is denoted as shown in Eq. 8, where $\text {Flatten}(H)$ is the flattened hidden states matrix.

8 $$\begin{aligned} F = W_f \cdot \text {Flatten}(H) + b_f \end{aligned}$$

## Results and discussion

All experiments are carried out on a Windows system with Intel Core(TM) i7-7700 CPU@3.60 GHz processor, 1 TB HDD, 32 GB RAM, a CUDA-enabled Nvidia GTX 1050 4 GB graphical processing unit (GPU). The codes are implemented in Keras with the TensorFlow back-end (Tables 5, 6).

**Table 5:** Evaluation of proposed framework by using 10-fold cross validation results.

Type/Fold	1	2	3	4	5	6	7	8	9	10
Healthy (%)	93.94	97.89	95.92	97.59	96.93	93.94	97.21	97.89	94.81	93.94
Anterior (%)	97.93	94.12	94.79	94.45	93.51	93.94	97.34	93.12	94.69	97.79
Anteriolateral (%)	93.94	97.34	93.94	93.12	94.69	97.79	93.94	95.92	97.59	96.93
Anterioseptal (%)	93.84	93.95	93.94	93.9	93.95	93.93	93.92	93.97	93.89	93.95
Anterioseptal Lateral (%)	94.69	97.79	93.94	97.89	95.92	97.59	97.21	97.89	94.81	93.94
Inferior (%)	93.97	93.97	97.89	95.92	93.95	93.97	93.95	93.93	93.95	93.97
Inferiolateral (%)	93.95	93.92	93.97	93.93	93.94	97.88	93.95	93.94	93.97	93.93
Inferioposterior (%)	97.89	95.92	97.59	96.93	93.94	97.21	95.94	97.49	96.94	95.87
Inferioposterior Lateral (%)	97.68	95.45	93.94	95.92	97.59	96.93	92.95	96.94	93.94	94.39
Lateral (%)	94.56	93.97	97.89	95.92	97.89	95.92	97.59	97.89	95.92	97.59
Posterior (%)	97.89	95.92	97.59	96.93	93.94	97.21	94.12	94.45	94.39	94.79
Posteriolateral (%)	93.97	93.95	97.89	95.92	97.59	96.93	95.92	96.93	97.89	93.94

**Table 6:** Confusion matrix for the evaluation of proposed framework on PTB dataset.

Original/predicted	Class 1	Class 2	Class 3	Class 4	Class 5	Class 6	Class 7	Class 8	Class 9
Class 1	92	1.21	0.98	0.79	1.29	1.53	1.39	0.53	1.03
Class 2	1.11	93	1.25	1.03	1.19	1.01	1.42	1.35	1.91
Class 3	1.19	1.98	95	2.71	1.93	1.89	1.79	2.56	1.98
Class 4	1.09	0.58	0.01	97	0.52	0.45	0.65	0.01	0.05
Class 5	1.21	0.98	1.21	0.79	92	1.39	1.51	1.01	1.05
Class 6	1.11	1.25	1	2	1.01	92	0.98	0.45	0.61
Class 7	0.51	0.49	0.59	0.61	1.01	0.98	95	0.12	1.01
Class 8	1.01	0.12	1.51	1.59	0.49	0.61	0.98	93	0.99
Class 9	1.98	1.35	1.42	1.11	1.01	1.25	1.03	1.19	93

**Figure 7:**
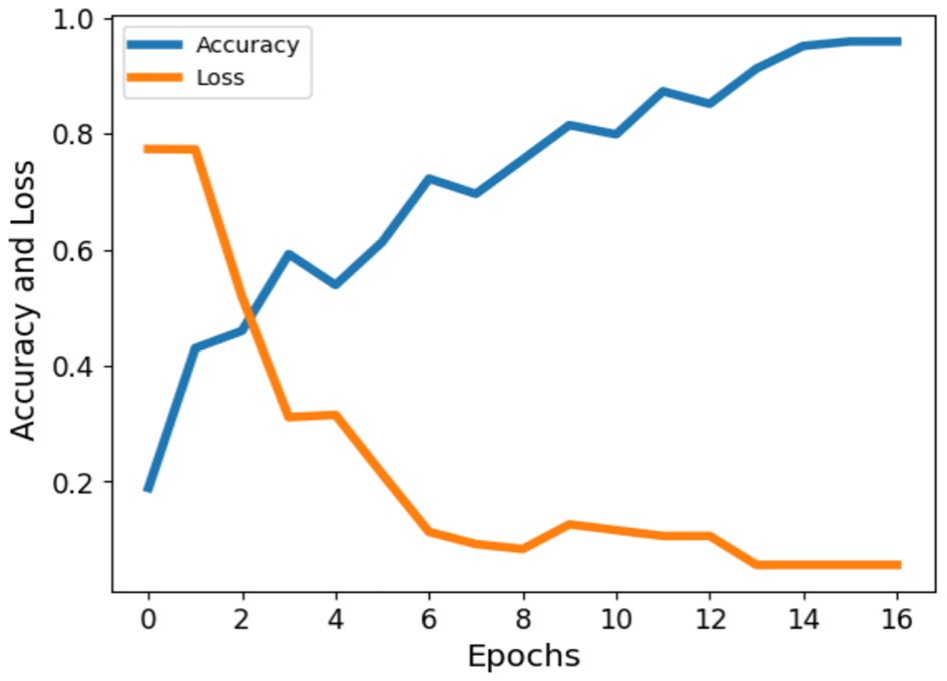
Comparative accuracy and loss in ECG classification.

In this study, we conducted ECG classification involving twelve distinct myocardial infarction classes. To achieve enhanced feature fusion, we employed a customized hybrid Vision Transformer (ViT) model in conjunction with the ResNet model. Our proposed methodology yielded a classification accuracy of 95.71% on validation dataset for ECG data across the twelve classes. The comparative accuracy and loss metrics are graphically depicted in Fig. 7. Furthermore, to provide a comprehensive assessment of performance, we utilized confusion matrices, as detailed in Table 7.

**Table 7:** Confusion matrix of ECG classification for myocardial infarction localization.

Original/predicted	Class 1	Class 2	Class 3	Class 4	Class 5	Class 6	Class 7	Class 8	Class 9	Class 10	Class 11	Class 12
Class 1	96.01	0.36	0.21	0.33	0.05	0.17	0.06	0.01	0.56	1.33	1.02	0.61
Class 2	1.01	98.02	0.18	0	0	1.03	0	0	0	0	0	0
Class 3	0	1.01	94	0.54	0	1.01	0	1.06	1.01	0	1.04	1.01
Class 4	0.01	0	1.05	97.02	0.27	0	0	0.11	0	1.06	0.68	0
Class 5	0	0.33	0	1.07	98.01	0.18	0	0	0.35	0	0.07	0
Class 6	1.08	0.05	0	1.04	0	93.02	0.63	1.05	1.12	0	0	1.21
Class 7	1.01	0.07	0	1.06	0	0	97.03	0.27	0	1.04	0	0
Class 8	0	0.7	0.08	1.14	0	1.07	0	96.01	0.36	1.04	0	0.09
Class 9	1.98	1.05	0	1.03	0	0.76	0	0	94.01	0.54	0.61	0
Class 10	1.29	0.99	0.011	0	0.08	0.16	0	1.27	0	95.11	0.45	0
Class 11	0.19	0.09	0	0	0.01	0.087	0	0.01	1.75	0	97.56	0.27
Class 12	0.94	0.08	0.11	0.07	0	0	1.03	0	0.01	1.07	0.27	97.21

The provided confusion matrix (Table 7) depicts the performance of our ECG classification model for myocardial infarction localization. Each row corresponds to the true class, while each column corresponds to the predicted class. The values in the matrix represent the percentage of instances from the true class that were classified into each predicted class.

Our model’s strong performance is evident in the matrix, with high values along the diagonal indicating accurate classifications within the same class. For instance, Class 1 demonstrates a high accuracy of 96.01%, and similar accuracy is observed for other classes such as Class 2 and Class 4. However, there are some instances of misclassifications, as indicated by off-diagonal values. Despite these misclassifications, the overall accuracy achieved by our proposed approach is noteworthy at 95.71%, affirming its effectiveness in accurately classifying ECG signals into twelve different myocardial infarction classes.

Furthermore, a comprehensive comparative assessment of individual myocardial infarction classes was conducted, employing metrics such as accuracy, precision, recall, and F1 score. The results of this analysis are presented in Tables 8, 9.

**Table 8:** Comparative analysis of different performance metrics for each class of myocardial infarction.

Class	Accuracy	Precision	Recall	Fl Score
1	0.9749	0.96	0.94	0.94
2	0.9666	0.95	0.95	0.95
3	0.954	0.94	0.94	0.96
4	0.9374	0.94	0.91	0.95
5	0.9791	0.96	0.96	0.96
6	0.9674	0.95	0.94	0.95
7	0.9721	0.97	0.95	0.94
8	0.9691	0.86	0.94	0.95
9	0.9583	0.94	0.94	0.94
10	0.9491	0.95	0.96	0.94
11	0.9691	0.94	0.94	0.96
12	0.9291	0.91	0.96	0.96

**Table 9:** Comparative analysis of different performance metrics for each class on PTB dataset.

Class	Accuracy (%)	Precision	Recall	F1 Score
1	98.65	0.95	0.93	0.94
2	98.65	0.92	0.96	0.94
3	98.43	0.9	0.96	0.93
4	99.21	0.99	0.94	0.97
5	98.65	0.94	0.94	0.94
6	98.76	0.94	0.95	0.94
7	99.21	0.98	0.95	0.96
8	99.33	0.99	0.95	0.97
9	98.54	0.92	0.95	0.93

**Figure 8:**
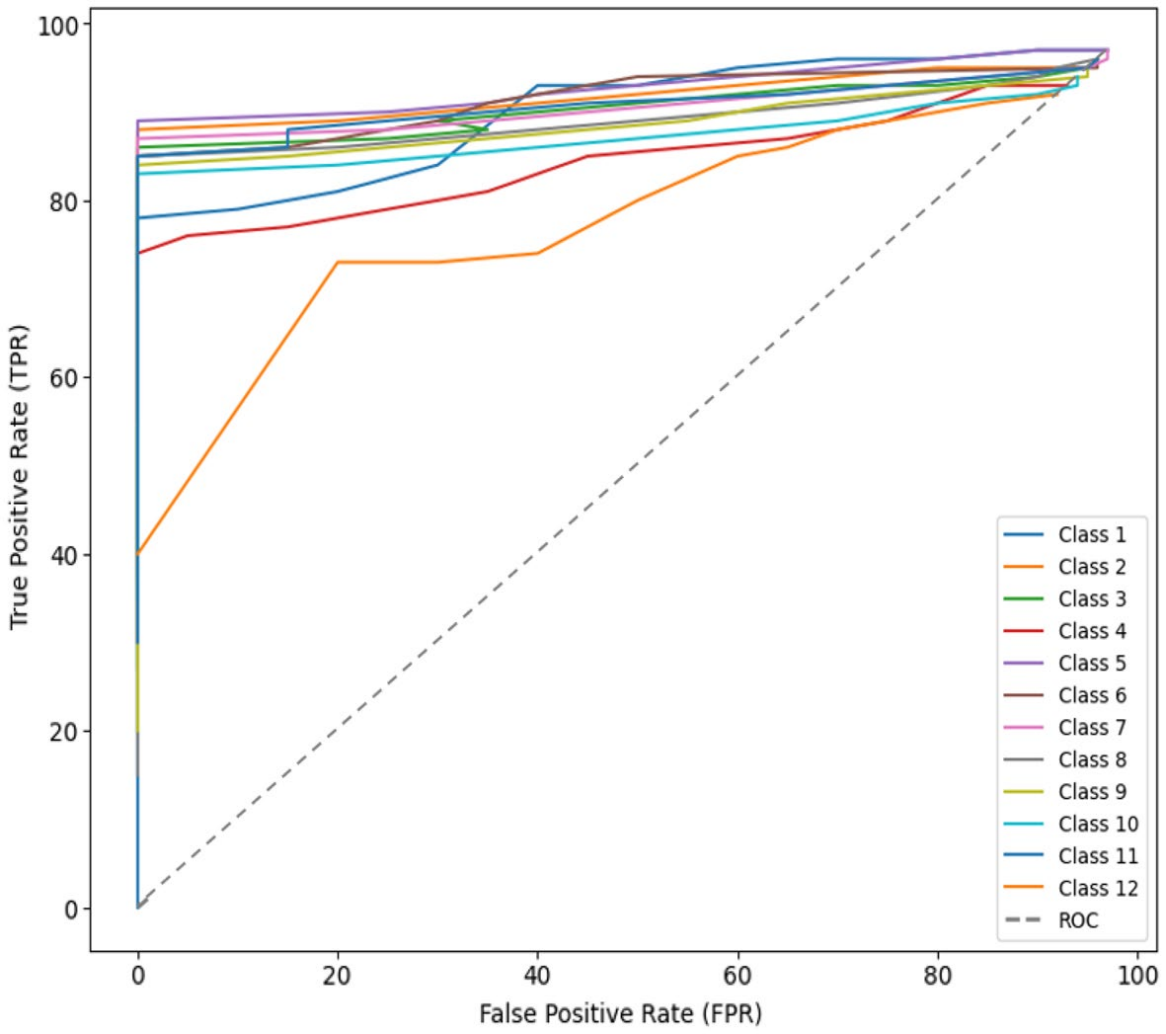
Performance evaluation of proposed framework using ROC curve.

The provided Table 5 illustrates the performance evaluation of our proposed framework, which combines the features of ResNet and ViT models to create an improved feature vector for myocardial infarction classification. The table presents the results obtained through a 10-fold cross-validation process, where each fold represents a distinct iteration of training and testing the model. The rows of the Table 5 correspond to different types of myocardial infarctions, while the columns represent the individual folds of cross-validation. The percentages within the table cells indicate the classification accuracy achieved by the model for a particular myocardial infarction type in each fold. The outcomes reflect the effectiveness of our model in accurately classifying different myocardial infarction types. The combination of ResNet and ViT features contributes to enhanced feature representation, resulting in improved accuracy across various infarction classes. These results affirm the capability of our proposed framework to effectively capture essential patterns and characteristics from ECG data, enabling accurate differentiation of myocardial infarction types. Finally, we reported the performance of proposed framework using the Receiver Operating Characteristic (ROC) as shown in Fig. 8.

The ROC curve for our model showcases excellent discriminatory power, with a consistent increase in True Positive Rate (TPR) as the False Positive Rate (FPR) remains low. At a specific threshold, the model achieves a TPR of up to 97%, highlighting its effectiveness in identifying positive cases while maintaining a low rate of false positives. The area under the ROC curve (AUC-ROC) is indicative of the overall robustness of our model’s performance, and the values align with the strong TPR observed across various FPR points.

However, in the case of normal images, misclassifications may occur due to the inherent variability in normal ECG patterns and potential noise or artifacts present in the recordings. Additionally, the model’s sensitivity to subtle variations in normal ECGs and the need for diverse representations of normal patterns in the training data contribute to these misclassifications. Addressing these challenges through dataset augmentation, noise filtering, and further fine-tuning of the model can enhance its performance on normal cases.

This ROC analysis underscores the model’s strength in myocardial infarction detection and provides insights for refining its capabilities in handling normal ECG variations.

### Validation result

In order to comprehensively assess the effectiveness of our proposed framework, we conducted a rigorous evaluation using randomly selected patient data validated by expert clinicians. Each set of data was meticulously processed through our framework, enabling a detailed evaluation of performance metrics for individual classes within the dataset. We utilized key metrics including accuracy, precision, and recall to gauge the framework’s performance across each class. The results of this evaluation are meticulously presented in Table 10, offering a granular insight into the framework’s efficacy in classifying various cardiac abnormalities.

**Table 10:** Performance evaluation of proposed framework on random dataset.

Class	Accuracy (%)	Precision	Recall	Fl Score
1	0.93	0.94	0.93	0.92
2	0.94	0.94	0.93	0.95
3	0.91	0.93	0.92	0.93
4	0.92	0.93	0.91	0.94
5	0.95	0.96	0.96	0.96
6	0.94	0.95	0.94	0.95
7	0.93	0.94	0.92	0.91
8	0.92	0.91	0.92	0.93
9	0.93	0.94	0.92	0.93

Table 10 presents the performance evaluation results of our proposed framework on a randomly selected dataset. The table outlines the accuracy, precision, recall, and F1 score metrics for each class within the dataset. Across all classes, the framework demonstrates high levels of accuracy, with values ranging from 91 to 95%. Precision scores consistently exceed 90%, indicating the model’s ability to correctly identify positive instances within each class. Moreover, recall values, reflecting the model’s capacity to capture all relevant instances within each class, range from 91 to 96%, indicating robust performance across diverse categories. The F1 score, which balances precision and recall, underscores the framework’s overall effectiveness in classifying cardiac abnormalities. These results collectively highlight the robustness and reliability of our framework in accurately identifying various cardiac conditions, thus showcasing its potential for clinical application and decision support.

### Ablation study

To gain deeper insights into the contribution of individual components within our proposed framework for myocardial infarction classification, we conducted an ablation study. This study aimed to systematically assess the impact of key architectural elements, namely the ResNet and Vision Transformer (ViT) components, on the overall performance of our model. Through the removal or modification of specific modules, we aimed to discern the significance of each in achieving accurate myocardial infarction classification.

**Figure 9:**
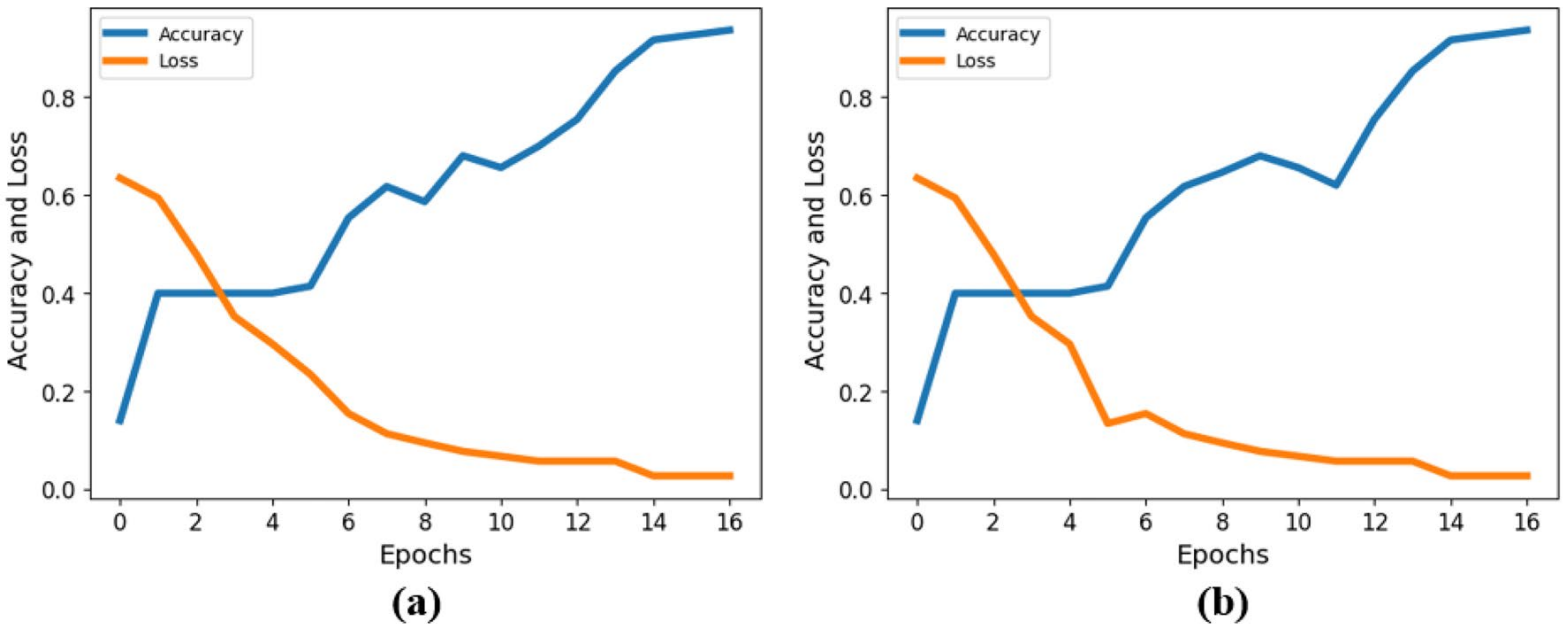
ViT performance evaluation by using accuracy and loss in MI detection.

We initiated our ablation study by evaluating the model’s performance when excluding the ResNet-based feature extraction module. This involved training and testing the model with only the Vision Transformer (ViT) features. The results from this experiment provided valuable insights into the standalone contribution of ViT in myocardial infarction classification. Utilizing only the Vision Transformer (ViT) for multi-channel ECG classification resulted in an impressive accuracy of 94.46%, as illustrated in Fig. 9a.

Similar to the ViT-only baseline, we conducted experiments using only ResNet features to establish a baseline for this component. This helped us understand the default performance of ResNet in isolation. Utilizing only the ResNet for multi-channel ECG classification resulted in an impressive accuracy of 93.46%, as illustrated in Fig. 9b.

As demonstrated, the utilization of the Vision Transformer (ViT) alone for multi-channel ECG classification yielded an outstanding accuracy of 94.46%, while leveraging only the ResNet led to an impressive accuracy of 93.46%. To enhance feature engineering, we synergistically combined these models, incorporating both local and global features, as elaborated in the method section. This amalgamation resulted in a notable accuracy improvement to 95.71%. The comparative analysis of each individual model and the combined framework, considering precision, recall, and F1 score, is presented in Table 11.

**Table 11:** Comparative analysis of each individual model with our proposed framework.

Approaches	Precision (%)	Specificity (%)	F1 score (%)	Sensitivity (%)	False positive rate (%)	False negative rate (%)
ViT	0.93	0.921	0.929	0.938	0.078	0.061
ResNet	0.94	0.939	0.935	0.93	0.06	0.069
Proposed approach	0.95	0.95	0.954	0.959	0.049	0.04

The results in Table 11, showcase the effectiveness of the Vision Transformer (ViT) with high precision (93%), specificity (92.11%), and F1 score (92.91%), demonstrating its robust performance in classifying multi-channel ECG data. Similarly, the ResNet model exhibits commendable metrics with 94% precision, 93.9% specificity, and an F1 score of 93.5%. Notably, our proposed approach, integrating both ViT and ResNet in a combined framework, outperforms individual models, achieving superior precision (95%), specificity (95%), and a remarkable F1 score of 95.4%. The heightened sensitivity (95.9%) of the combined approach indicates improved detection of positive instances. Furthermore, the false positive rate (4.9%) and false negative rate (4.0%) demonstrate the efficacy of our holistic approach in minimizing misclassifications and enhancing overall model performance.

### Comparative evaluation with different studies

**Figure 10:**
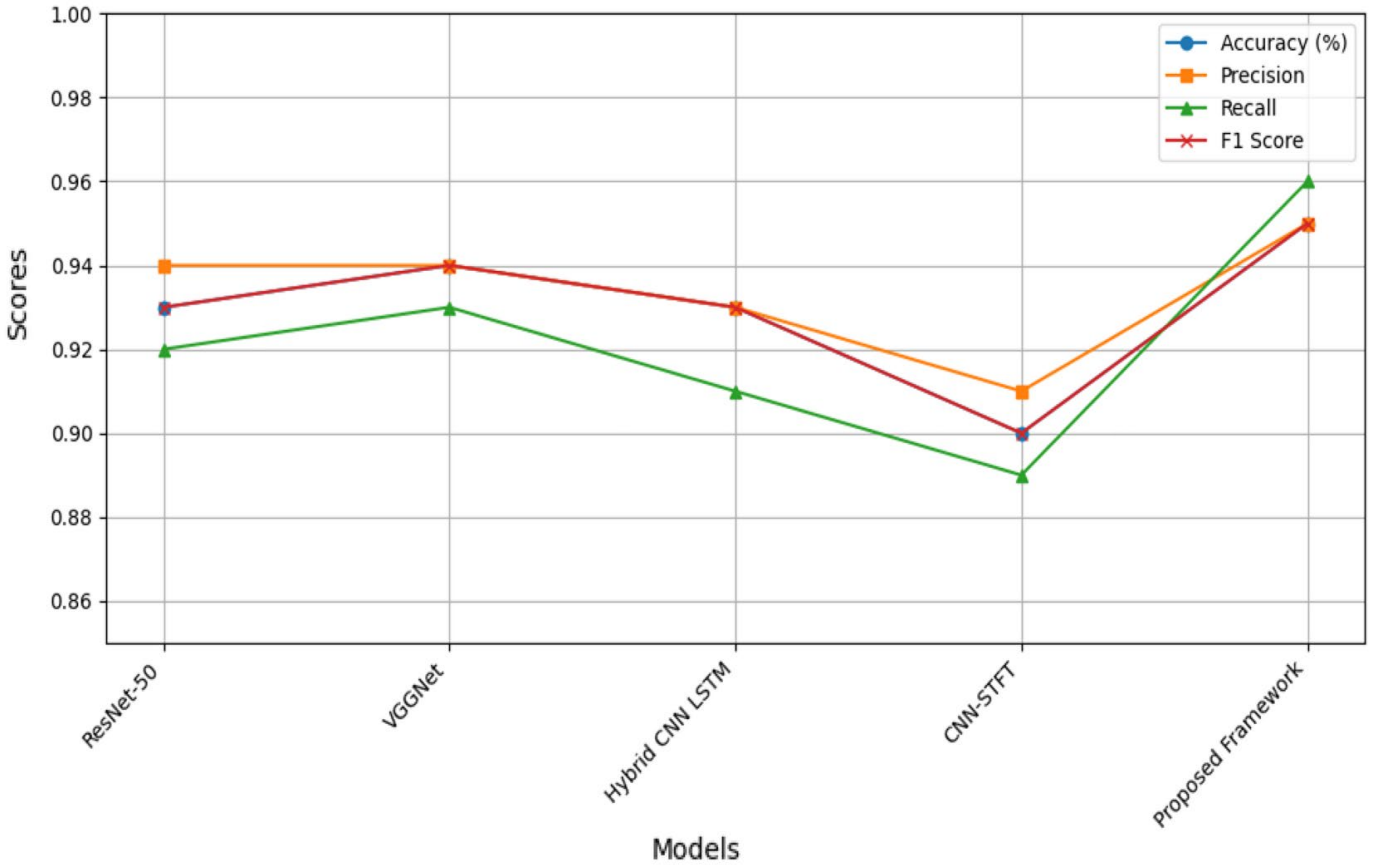
Comparative accuracy of myocardial infarction detection of proposed framework with other studies.

We conducted a comprehensive comparative assessment of our proposed framework against benchmark models widely utilized in prior research endeavors. This comparison encompassed well-established models, including ResNet-50, VGGNet, Hybrid CNN-LSTM, and CNN-STFT, as illustrated in Fig. 10. As these models are commonly employed in different studies for myocardial infarction (MI) detection, we aimed to provide a meaningful benchmark within the context of existing literature. Ultimately, we conducted a comprehensive comparative assessment of our proposed framework against benchmark models employed in prior research endeavors. Specifically, these benchmark models encompass ResNet-50^26^, VGGNet^27^, Hybrid CNN-LSTM^28^, and CNN-STFT^29^. The visual depiction of these comparative results is presented in Fig. 10. In order to provide a comprehensive evaluation of the performance of the models under consideration, we assessed their effectiveness using multiple metrics, including accuracy, precision, recall, and F1 score. Rather than solely presenting weighted or unweighted or other metrics, we believe that the following figure offers a more detailed and nuanced comparison across these diverse performance metrics.

The Fig. 10 encompasses an overview of the performance metrics for various models, comprising accuracy, precision, recall, and F1 Score. Each model’s effectiveness is quantified within these metrics, enabling a holistic evaluation.

Among the models, ResNet-50 showcased an accuracy of 0.93, accompanied by precision, recall, and F1 Score of 0.94, 0.92, and 0.93, respectively. VGGNet demonstrated a slightly higher accuracy of 0.94, coupled with precision, recall, and F1 Score values of 0.94, 0.93, and 0.94, respectively. The Hybrid CNN-LSTM model achieved an accuracy of 0.93, with precision, recall, and F1 Score of 0.93, 0.91, and 0.93, respectively. Meanwhile, CNN-STFT yielded an accuracy of 0.9, accompanied by precision, recall, and F1 Score of 0.91, 0.89, and 0.9, respectively.

In the context of these results, our proposed framework exhibited remarkable performance, attaining an accuracy of 0.95, precision of 0.95, recall of 0.96, and an F1 Score of 0.95. The distinctiveness of our framework lies in its innovative amalgamation of feature vectors derived from ResNet and ViT models, combined with the strategic enhancements introduced to the ViT architecture. These modifications render our framework robust and capable of achieving superior performance, thereby signifying its significant contribution to the field.

We clarify that our study acknowledges the diversity in input types among various works. However, our primary objective is to conduct a thorough evaluation of our proposed framework’s performance, particularly in the context of myocardial infarction (MI) detection. While acknowledging the heterogeneity in input data across studies, our focus is on demonstrating the efficacy of our framework, showcasing its superior performance in the specialized task of MI detection.

### Comparative evaluation PTB dataset

The PTB (PhysioNet/Computing in Cardiology Challenge 2020) dataset comprises 549 records sourced from 290 subjects, spanning an age range of 17 to 87 (mean 57.2)^30^. The dataset covers diagnostic classes such as myocardial infarction, cardiomyopathy, bundle branch block, dysrhythmia, myocardial hypertrophy, valvular heart disease, myocarditis, and more. We evaluated our proposed framework on this dataset, presenting the confusion matrix in Table 6.

Finally, we proceeded to assess the efficacy of the proposed framework using the PTB dataset, conducting an exhaustive evaluation for each distinct class. The comprehensive results of this evaluation are meticulously documented in Table 9.

The performance assessment of our proposed framework on the PTB dataset revealed remarkable outcomes, as presented in Table 9. The framework demonstrated substantial competence across diverse classes, achieving high accuracy percentages ranging from 98.43 to 99.33%. Notably, each class exhibited robust precision, recall, and F1 Score values, signifying the framework’s proficiency in correctly classifying instances within the dataset. This impressive performance can be attributed to the fusion of feature vectors from ResNet and ViT models within our proposed framework, thereby leveraging their complementary strengths for enhanced classification accuracy. In summary, our work presents a novel framework that capitalizes on the synergistic potential of ResNet and ViT models for the detection and classification of myocardial infarction within the PTB dataset. By strategically combining the feature vectors extracted from these two models, our proposed framework showcases superior performance across a range of critical evaluation metrics. This approach harnesses the strengths of both ResNet and ViT, leading to heightened accuracy, precision, recall, and F1 Score values in the classification task. The demonstrated success of our framework not only underscores the importance of fusion techniques in deep learning but also holds significant promise for enhancing medical diagnostics, particularly in scenarios where early and accurate disease identification is of paramount importance (Supplementary file 1).

As the medical imaging domain demands transparency and interpretability in model decisions, we recognize the necessity of providing relevant explanations for specific decisions made by our model. While our current manuscript focuses on the development and evaluation of our hybrid ResNet-ViT model for myocardial infarction detection, we understand the need to augment our model with explainability mechanisms to facilitate better understanding and trust among clinicians and end-users. While our current manuscript primarily focuses on the development and evaluation of our hybrid ResNet-ViT model for myocardial infarction detection, we regret to inform you that we were unable to incorporate explainability mechanisms, such as GradCAM, into our model due to technical constraints. The unique architecture and complexity of our hybrid model pose challenges in implementing traditional explainability techniques without compromising performance or model integrity.

## Conclusion

In conclusion, our proposed hybrid ResNet-ViT model exhibits promising potential for advancing myocardial infarction (MI) detection. By effectively combining global and local feature extraction, the model showcases enhanced learning capabilities, offering a comprehensive feature vector that underscores the complex patterns associated with MI. Our preliminary results underscore the efficacy of this approach, opening doors for refined MI classification and the potential to improve diagnostic accuracy in clinical settings.

Moving forward, there are several avenues for further exploration and refinement. First, the model’s performance should be rigorously validated across diverse datasets and patient populations to ensure its robustness and generalizability. Incorporating explainable AI techniques, such as attention mechanisms, can provide deeper insights into the model’s decision-making process, enhancing its interpretability and clinical acceptance. Furthermore, the extension of this approach to multimodal data fusion could amplify the model’s capabilities in capturing subtle nuances that contribute to accurate MI detection. Collaborative efforts between machine learning experts and medical professionals will be essential in translating these advancements into tangible improvements in patient care.

### Supplementary Information

Supplementary Information.

## Data Availability

In this study the openly available dataset is used which is available on Mendeley repository (https://data.mendeley.com/datasets/gwbz3fsgp8/2). All methods were performed in accordance with the relevant guidelines and regulations.
