# Supplementary material for: A hybrid ResNet-ViT approach to bridge the global and local features for myocardial infarction detection
Source: Sci Rep. 2024 Feb 22;14:4359. doi: 10.1038/s41598-024-54846-8 (PMC10883929; doi:10.1038/s41598-024-54846-8)
Supplement: Supplementary file 1 — Supplementary Information. [file 41598_2024_54846_MOESM1_ESM.pdf]

Bridging Global and Local Features: A Hybrid ResNet-ViT Approach for Myocardial Infarction Detection

Junaid Abdul Wahid[1,+], Xu Mingliang[1] , Muhammad Ayoub[2,*,+], Shabir Husssain[3,+], Lifeng Li[4] and Lei Shi[5]
[1]School of Computer Science and Artificial Intelligence, Zhengzhou University, Henan, 450001, China
[2]School of Computer Science and Engineering, Central South University, Changsha, 410017, Hunan, China
[3]School of Architecture, Harbin Institute of Technology, Shenzhen, 518055, Guangdong, China
[4]Department of Radiology, The Affiliated Changsha Central Hospital, Hengyang Medical school, University of South China, Changsha, 410017, China
[5]School of Cyberspace and Security, Zhengzhou University, Zhengzhou, Henan, 450001, China
[*]corresponding.ayoubncbae@gmail.com
[+]these authors contributed equally to this work

The inclusion criteria for the subjects in ECG

- Inclusion criteria encompass individuals aged $\geq$ 18 years.
- Eligible participants must exhibit a definitive diagnosis of acute ST-elevation myocardial infarction (STEMI), characterized by ST-segment elevation in a minimum of 2 contiguous leads. Additionally, individuals meeting at least one of the following criteria are considered:

  - Manifestation of myocardial ischemic symptoms, including chest tightness, chest pain, precordial squeezing, and retrosternal oppressive pain, which remain unresolved despite the administration of rapid-acting nitroglycerin or nitrate drugs. In elderly or diabetic patients less sensitive to pain, symptoms such as abdominal distension, nausea, and vomiting, after ruling out primary digestive system diseases, may also be indicative of insufficient organ perfusion linked to myocardial ischemia.
  - Elevated levels of cardiac injury biomarkers (troponin, creatine kinase isoenzymes, myoglobin) surpassing the upper threshold of the normal reference range by 99\% or more.

- Participants must have undergone emergency coronary angiography within 12 hours from the onset of acute myocardial infarction, with the results of angiography confirming the presence of coronary artery disease.
- Essential for inclusion is the availability of comprehensive 12-lead electrocardiogram data at the time of symptom onset.

The exclusion criteria for the subjects in ECG

- Poor electrocardiogram quality, which impedes the accurate measurement of parameters or leads that are missing.
- The presence of pacing rhythm on the electrocardiogram attributed to the insertion of a permanent pacemaker.
- Second-degree or higher degree atrioventricular block.
- Pre-existing permanent or intermittent left bundle branch block or left posterior fascicular block.
- Patients who have undergone thrombolytic therapy in the emergency department.
- Time from symptom onset to guidewire passage through the culprit vessel surpassing 12 hours.
- Coronary angiography revealing left main stem disease, three-vessel coronary artery disease, or an inability to identify the culprit vessel.
- Patients who have developed ventricular aneurysm subsequent to a previous myocardial infarction.
- Early repolarization syndrome.
- Cardiac conditions such as pericarditis and myocarditis that may induce ST-segment elevation.
- Medical conditions such as intracranial hemorrhage, pneumothorax, and aortic dissection that can result in secondary ST-segment elevation.
- Severe electrolyte imbalances.
- The use of digitalis medications.
